# The Impact of a Videogame-Based Pilot Physical Activity Program in Older Adults with Schizophrenia on Subjectively and Objectively Measured Physical Activity

**DOI:** 10.3389/fpsyt.2015.00180

**Published:** 2015-12-21

**Authors:** Heather Leutwyler, Erin Hubbard, Bruce Cooper, Glenna Dowling

**Affiliations:** ^1^Physiological Nursing, University of California San Francisco, San Francisco, CA, USA; ^2^School of Nursing, University of California San Francisco, San Francisco, CA, USA

**Keywords:** schizophrenia, physical activity, videogame

## Abstract

**Objectives:**

The purpose of this report is to describe the impact of a videogame-based pilot physical activity program using the *Kinect for Xbox 360* game system (Microsoft, Redmond, WA, USA) on physical activity in older adults with schizophrenia.

**Methods:**

In this one group pre-test, post-test pilot study, 20 participants played an active videogame for 30 min, once a week for 6 weeks. Physical activity was measured by self-report with the Yale Physical Activity Survey and objectively with the Sensewear Pro armband at enrollment and at the end of the 6-week program.

**Results:**

There was a significant increase in frequency of self-reported vigorous physical activity. We did not detect a statistically significant difference in objectively measured physical activity although increase in number of steps and sedentary activity were in the desired direction.

**Conclusion:**

These results suggest participants’ perception of physical activity intensity differs from the intensity objectively captured with a valid and reliable physical activity monitor.

## Introduction

Older people with schizophrenia tend to spend the majority of their time in sedentary activity (1). Prolonged sedentary activity is associated with various negative health outcomes including poor physical fitness (2). The negative impact of sedentary activity is problematic for any adult, but is particularly burdensome for older adults with schizophrenia. People with schizophrenia have a higher mortality rate than the general population and sedentary behavior contributes to this mortality gap (3, 4). Even small reductions in sedentary behavior, such as short activity intervals of 10 min or less, may positively influence physical and mental health (5).

Despite the obvious need for physical activity programs to decrease sedentary time in older adults with schizophrenia, few interventions exist. Most of the physical activity research in serious mental illness has focused on younger to middle-aged adults (6). In a 2010 Cochrane review, the efficacy of three physical activity RCTs (e.g., walking, weight training) in people with severe mental illness (SMI) was evaluated (6). The authors concluded that the impacts of these programs on health outcomes were mixed, but some studies provided evidence of improved fitness. Two physical activity RCTs for people with SMI published after the Cochrane review also produced mixed outcomes. One study provided evidence of a positive impact on weight change but did not report information about impact on physical activity (7). Another study showed a positive impact on self-reported physical activity levels (8).

Not only do we need effective physical activity programs for older adults with schizophrenia but we also need programs that will successfully engage older adults with schizophrenia (6, 9). Videogame-based exercise may be one way to promote physical activity in this vulnerable population (10, 11). Videogames with an interface that requires physical exertion to play, such as the *Kinect for Xbox 360* game system (Microsoft, Redmond, WA, USA), promote physical activity (11, 12). Participants use their body to control the game with the *Kinect’s* full-body tracking sensor system that recognizes the participant’s body and mirrors those movements in the game.

We designed and tested a videogame-based physical activity program designed to keep older adults with schizophrenia engaged and motivated to participate in physical activity. (10, 11) Consistent with previous findings from videogame research, (13) as our participants became more involved and successful with active videogames, they developed skills that made it easier to engage in activity (10). One of the study objectives was to examine the impact of the program on physical activity in participants’ every day life. The purpose of this report is to describe the impact of a videogame-based physical activity program using the *Kinect for Xbox 360* game system (Microsoft, Redmond, WA, USA) on frequency of vigorous physical activity in older adults with schizophrenia.

## Materials and Methods

### Design

A one-group pre-test, post-test design was used to examine the impact of our pilot program on physical activity. Institutional review board approval was obtained from the University’s Committee on Human Research. Anonymity and confidentiality were maintained according to their guidelines.

### Participants and Settings

Inclusion criteria were that participants be: (1) at least 55 years of age or older; (2) diagnosed with schizophrenia or schizoaffective disorder; and (3) competent to consent based on an evaluation of their comprehension of the consent form. A convenience sample of participants from four different facilities were recruited by facility staff and referred to the researchers. Study fliers were also posted. Patients with a history of a prior myocardial infarction, uncontrolled hypertension, history of angioplasty, history of angina, and/or use of nitroglycerin to treat angina were excluded.

### Procedures

Recruitment and data collection began in May 2012 and concluded in June 2013. The principal investigator and her research staff facilitated the weekly exercise sessions. Once a week for 6 weeks, participants played an active videogame, using the *Kinect for Xbox 360* game system (Microsoft, Redmond, WA, USA), for 30-min. Although current recommendations for adults are to engage in moderate intensity physical activity 150 min each week, (14) the focus of this pilot study was to determine feasibility, acceptability, and adherence to the program. Therefore, we chose a short frequency and duration for our program to first establish participant participation in the program.

At each weekly session, participants choose from a variety of games and were encouraged to use different games each week. Off-the-shelf videogames played included: *Kinect Sports* (e.g., bowling, golf, skiing, darts), *Kinect Carnival Games*, *Kinect Dance Central 2*, *Kinect Adventures*, and *Kinect Your Shape Fitness Evolved*. Games played most often were: bowling, dance, carnival games, skiing, Tai Chi (from the Fitness game), baseball, darts, golf, river rafting, and 20,000 leaks under the sea.

Participants engaged in the program in groups of three to four at a time. The program took place at the facility the person attended: an outpatient community treatment center, a locked mental health facility, a transitional residential facility, or a skilled nursing facility.

Each participant had an appropriate amount of space in order to achieve full range of motion. Approximately 6 feet of free space between the participant and the *X-Box Kinect* sensor was needed. The games offered a variety of levels and each group started off at the beginner level. Participants were taught warning signs to be aware of while exercising (e.g., shortness of breath, dizziness), were encouraged to discontinue the game if they noticed any exercise warning signs and to notify the research staff.

### Measures

A demographic questionnaire obtained information on age, gender, race, smoking status, and living situation at enrollment.

#### Physical Activity

Subjective assessment of physical activity was assessed with the Yale Physical Activity Scale (YPAS). (15) The YPAS is based on self-report and measures five activity dimensions (Vigorous activity, Leisurely walking, Moving on feet, Standing, and Sitting) that occur during a typical week over the past month. Eight summary scores are calculated that include: total time spent per week in all physical activities, total energy expenditure in kcal per week, five individual indices for the activity dimensions, and a total activity dimension index. Indices are calculated from the frequency, duration, and intensity of activities. Preliminary data supports the use of YPAS as a self-report measure of physical activity in older adults with schizophrenia (16). The YPAS was completed at enrollment into the study and upon completion of the final group at week 6.

Objective assessment of physical activity was measured with the SenseWear Pro armband™ (SWA; BodyMedia Inc., Pittsburgh, PA, USA). Participants wore the device for 7 days between week 1 and week 2 and again between week 5 and week 6 over the left triceps muscle at all times except during bathing or water activities. This device samples data from a heat-flux sensor, a galvanic-skin-response sensor, a skin-temperature sensor, a near-body-temperature sensor, and a bi-axial accelerometer. Previous research doucmented that the SWA accurately estimates energy expenditure in older adults. (17) Outcome variables measured with this instrument included daily steps taken during a week and weekly hours of physical activity categorized as sedentary [0–2.9 metabolic equivalents (METS)], moderate (3–5.9 METS), vigorous (6–8.9 METS), and very vigorous (≥9 METS). The data can be reduced to a single number in order to determine if activity levels improved during the course of the program. Participants were fitted with the SWA and informed about the activities the device would monitor during data collection. Participants were reminded to take the SWA off only when showering or performing other activities during which the device might get wet. Additionally, they were given an information sheet that described proper placement and contact information for study staff if they had questions at any time. The devices were returned at the next videogame session. If the participant was unable to attend, arrangements were made for a convenient time and location for retrieval.

Adherence was measured with a count of sessions attended and with total minutes attended out of the possible total minutes of attendance. Total possible minutes for the six sessions were 180. The PI or research assistant (RA) logged the participant’s attendance at each session. The PI or RA monitored participants throughout the session in order to determine the number of minutes attended as participants were allowed to leave at any point during the group.

### Data Analysis

Statistical analyses were performed using STATA version 13. Descriptive statistics and frequency distributions were generated for sample characteristics. Change in self-reported vigorous physical activity with the YPAS was analyzed with a non-parametric bootstrapped repeated measures *t*-test using bias-corrected and accelerated confidence intervals with 5,000 repetitions to compensate for the non-normal distribution of difference scores.

Change in objectively measured physical activity from week 1 to week 6 was evaluated with a change score coded as a dichotomy, with positive change (i.e., increase) coded 1 and negative (i.e., decrease) or no change coded as 0, subtracting the total amount of activity measured during week 1 from week 6. This approach accommodated the extreme variation in the small number of reports (e.g., SD almost seven times greater than the mean for change in sedentary hours reported) by allowing a simple binomial test for change.

## Results

A total of 20 participants that took part in the study completed the physical activity assessments and are included in the analyses. Sociodemographic and clinical characteristics are presented in Table 1. The majority of participants were male and the average age was 60 years. More than half of the patients were current or previous smokers.

**Table 1:** **Clinical characteristics**.

Characteristic	Mean or ratio (SD)
Age (in years)	60.3 (4.4)
Male	16/20
Smoking status
Current	11/20
Past	6/20
Never	3/20
Residence
With family	1/20
Apartment with roommate	4/20
Apartment alone	1/20
Board and care	9/20
Hotel	1/20
Psychiatric facility	4/20
Race	
Caucasian	12/20
African American	3/20
Latino	1/20
Asian	1/20
Native American	1/20
Other	2/20

The mean number of groups attended was 5.6 out of 6 total (SD = 0.8) and the mean total minutes attended were 169 out of 180 total (SD = 23.7). Seventy percent of participants (*n* = 14) had perfect attendance (i.e., attended six out of six sessions), four participants attended five sessions, one participant attended four sessions, and one participant attended three sessions.

Vigorous physical activity was defined as any activity that lasted at least 10 min and caused large increases in breathing, heart rate, or leg fatigue, or caused the person to perspire. At enrollment in the study, 13 participants reported no vigorous activity, 4 participants reported vigorous activity 1–3 times a month, 1 participant reported vigorous activity 1–2 times per week, 1 participant reported vigorous activity 3–4 times per week, and 1 participant reported vigorous activity 5 or more times per week. At completion of the study, nine participants reported no vigorous activity, four participants reported vigorous activity one to three times a month, one participant reported vigorous activity one to two times per week, three participants reported vigorous activity three to four times per week, two participants reported vigorous activity five or more times per week, and one did not know. With the non-parametric bootstrapped repeated measures *t*-test using bias-corrected and accelerated confidence intervals (95% CI) and alpha equal to 0.05, we detected a significant increase in frequency of self-reported vigorous physical activity from enrollment to the end of the program for an average increase of 0.9 points (95% CI: 0.35–2.45). This difference constitutes a standardized effect of 0.46 [Cohen’s *d*; (18)], which is in the “medium effect size” range.

Fifteen of the 20 participants wore the SWA. Five participants chose not to wear the SWA. As indicated in Table 2, 10 participants increased the number of steps taken during the period between enrollment to program completion and 5 showed no increase. Nine participants had a decrease in the amount of time spent in sedentary activity while six had no increase. Eight participants increased the amount of time spent in moderate activity, and seven showed no increase. We did not detect any vigorous or very vigorous activity. None of these differences were statistically significant in this small sample, although increase in the number of steps taken (67% increased), and the reduction in sedentary activity (61% decreased) were in the desired direction.

**Table 2:** **Objective physical activity results**.

Change in physical activity week 1–6	Number of participants
Steps
No increase	5
Increase	10
Sedentary hours
No increase	6
Decrease	9
Moderate hours
No increase	7
Increase	8

## Discussion

Our videogame-based physical activity program is the first in the literature to show a significant positive impact on the amount and frequency of physical activity in older adults with schizophrenia. While participants self-reported significantly more vigorous physical activity, we did not find a statistically significant difference in objectively measured physical activity. However, the majority of participants increased their number of steps and spent less time in sedentary activity at program completion. It was interesting to find that although we did not detect any vigorous or very vigorous activity with our monitors, participants self-reported an increase in vigorous activity at program completion. Further research need to be done to better understand the differences between perceived and actual levels of physical activity in this population. It is possible that when a sedentary patient begins an exercise program, the patient perceives the activity to be more vigorous than what is objectively recorded by an activity monitor. These data also suggest that our program is an ideal way to begin a physical activity program because it allows participants to begin activity in a safe and feasible manner while providing the opportunity to decrease sedentary activity and increase daily steps taken.

Interestingly, Lindamer et al. (16) also found that older patients with schizophrenia self-reported vigorous activity differed from objectively measured vigorous activity. Soundy et al. (19) conducted a systematic review to identify and synthesize the evidence about levels of physical activity in adults with serious mental illness. Not surprisingly, the authors found that patients spent more time per week in sedentary activity and less time per week in moderate and vigorous activity compared to healthy controls. Physical activity was measured in a variety of ways in these studies, including both actigraphy and self-report with the IPAQ. These authors encourage the use of both rigorous self-report questionnaires as well as accelerometers to capture the range of physical activity. Our results also highlight the importance of evaluating physical activity with both objective and subjective measures in order to capture the full spectrum of activity and inactivity.

Similar to our results, Bartels et al. (8) found in their physical activity program (In SHAPE) for adults of all ages with serious mental illness, that intervention participants self-reported twice as much moderate to vigorous activity compared to controls. The intervention group also had significantly greater total MET minutes of vigorous activity. Their measure of physical activity was self-report using the International Physical Activity Questionnaire (IPAQ). In contrast to the YPAS, the IPAQ only assesses moderate and vigorous physical activity. Other researchers have suggested that the YPAS may be more likely to capture light activity than an objective physical activity monitor (16). The intervention for the In SHAPE program consisted of 1 year of weekly sessions with a fitness trainer plus a fitness club membership. This approach may have also offered a way for participants to begin a physical activity program in an individualized way that was safe and approachable.

Recent research suggests that promoting light intensity physical activity may be a feasible approach to ameliorate the deleterious health consequences associated with sedentary behavior (20). Low levels of physical activity in combination with prolonged sitting time are associated with negative health outcomes (20, 21). Greater sedentary time has been associated with an increased risk for all-cause mortality in the general population (22). Our program may be an avenue to break up prolonged sitting time because it is a feasible gentle physical activity program to begin with that can be adapted to become more strenuous as participants become adept at both playing the games and more comfortable with engaging in physical activity.

Our study has several limitations. Since this was a pilot feasibility study, participants engaged in the program only once a week for 30 min. Perhaps with more frequent activity, the impact on objective measures would have been more pronounced. In addition, the majority of the participants were male. In order to determine if the program is helpful for both men and women, in future work we will attempt to recruit and enroll more females. The lack of a control group makes it difficult to fully understand the impact of the program on participants’ physical activity levels. One of the purposes of the study was to determine which games participants preferred however this could have influenced individual participants’ activity levels differently. In future work, we plan to standardize the games played during the course of the study so that participants all experience the same games for equal amounts of time. Despite the limitations, our study provides important preliminary data about the initial efficacy of a novel videogame-based physical activity program that has potential to improve physical activity and health outcomes in people with schizophrenia.

Our results indicate that participants have a positive attitude toward videogame-based physical activity. Although participants’ perception was that of engaging in vigorous activity, our objective monitor did not detect vigorous activity. However, objectively measured physical activity did increase, showing a trend toward improvement. With a larger sample, more frequent exercise, and increased exercise duration, we hope to see a significant improvement in objectively measured activity. For example, with 40 participants, the medium effect size for change in sedentary time would be statistically significant with alpha = 0.05 (two-sided) and power = 0.80. The results from this study represent an important “first step” toward the creation of a novel and effective physical activity program for older adults with schizophrenia that may be easily incorporated into the daily routine of mental health facilities.

## Author Contributions

HL, EH, BC, and GD made substantial contributions to the conception of the work, analysis and interpretation of the data for the work. HL and EH made significant contributions to the acquisition of the data. HL drafted the work. HL, EH, BC, and GD revised the work critically for important intellectual content, approved the final version, and agree to be accountable for all aspects of the work.

## Conflict of Interest Statement

The authors declare that the research was conducted in the absence of any commercial or financial relationships that could be construed as a potential conflict of interest. The reviewer Jerome Favrod and handling Editor Yasser Khazaal declared a current collaboration and the handling Editor states that the process nevertheless met the standards of a fair and objective review.
